# Experimental and Analytical Investigation of Flexural Behavior of Carbon Nanotube Reinforced Textile Based Composites

**DOI:** 10.3390/ma16062222

**Published:** 2023-03-10

**Authors:** Emrah Madenci, Yasin Onuralp Özkılıç, Ceyhun Aksoylu, Muhammad Rizal Muhammad Asyraf, Agusril Syamsir, Abu Bakar Mohd Supian, Bobrynina Elizaveta

**Affiliations:** 1 Department of Civil Engineering, Necmettin Erbakan University, 42090 Konya, Turkey; 2 Department of Civil Engineering, Konya Technical University, 42090 Konya, Turkey; 3 Engineering Design Research Group (EDRG), Faculty of Mechanical Engineering, Universiti Teknologi Malaysia, Johor Bahru 81310, Johor, Malaysia; 4 Centre for Advanced Composite Materials (CACM), Universiti Teknologi Malaysia, Johor Bahru 81310, Johor, Malaysia; 5 Institute of Energy Infrastructure (IEI), Universiti Tenaga Nasional, Jalan IKRAM-UNITEN, Kajang 43000, Selangor, Malaysia; 6 World-Class Research Center “Advanced Digital Technologies”, State Marine Technical University, 190121 Saint-Petersburg, Russia

**Keywords:** CNT, FRP, composite, carbon, CFRP

## Abstract

In this study, the main goal of this study was to understand the effect of carbon nanotube (CNT) additives on the elastic behaviors of textile-based composites. The materials have three phases: carbon fiber fabric, epoxy matrix, and carbon nanotubes. Different weight fractions of CNTs were used (0% as a reference, 0.3%). Mechanical tests were performed, such as tension and three-point bending beam tests. In addition, the composite material damages were examined in detail. The experimental results show that the samples with CNT carried 9% and 23% more axial tensile force and bending capacity on average than those with NEAT. Besides, it was understood that adding 0.3% by weight of MWCNT increases the tensile modulus by approximately 9%. Finally, the mechanical tensile and bending tests are supported by analytical solutions successfully applied in the literature.

## 1. Introduction

Due to their advanced features, high specific strength, resistance to corrosion, and tailored designs, multilayer fiber-reinforced polymer (FRP) composite structures have become more prevalent in the aerospace, mechanical, civil, and automotive industries [1,2,3,4,5]. A composite material is a system of materials made up of a matrix and reinforcing fillers. These two components work together to produce unique features neither could have on their own [6,7,8,9]. In polymer matrix composites, fibers with fine diameters (carbon, glass, boron, aramid, Kevlar, and light carbon-based fibers) are used as the matrix and polymer (e.g., epoxy, polyester, urethane) as the binding agent. The reason for their widespread use can be attributed to their low price, high strength and easy production methods [10,11,12].

The capacity of FRPs in composite constructions is significantly influenced by the mechanical properties of the matrix [13,14,15,16]. The presence of the matrix will ensure that the load is evenly distributed among all fibers. It requires a tensile strength under tensile and shear loads, adhesion between the components (fiber and matrix), and a high shear strength capacity of the matrix. In the direction parallel to the fiber orientations (toward the length of the fibers), the mechanical properties of the matrix and the bond forces between the fiber and matrix have a significant role in determining the strength. The matrix is more flexible and less strong than the fiber. The design of composite constructions should take this aspect into account. If the shear strength of the matrix and the bond strength between the matrix and the fiber are very high, a crack can occur in the fiber or matrix to propagate in all directions without changing direction. In this case, since the composite acts as a brittle material, the rupture surface shows a clean and shiny structure. If the bond strength is too low, the fibers behave like a fiber bundle in the void, and the composite weakens. A crack in the transverse direction starting from the fiber or matrix can return to the fiber/matrix interface and progress in the fiber direction at a moderate bond strength. In this case, the composite exhibits a fibrous surface, such as the rupture of ductile materials.

CNT attracts attention as a cutting-edge material of this century due to its excellent physical, electrical, and chemical properties [17,18]. The mechanical strength of the CNT-reinforced polymer (CNTRC) composite increases significantly when only a tiny amount of CNT is added [19]. CNTRCs, used as content components in different engineering applications, have been developed as beam [20], plate [21,22], and shell [23] elements. Compared to conventional composites, CNTRCs ensure superior mechanical behaviors such as bending deformation, free vibration, and buckling [24,25,26,27]. Carbon nanotubes (CNTs) are also attractive substances with a wide range of uses. A CNT may be thought of as a one-dimensional molecular cylinder. Due to their high mechanical aspects and low density, CNTs have been recognized as attractive options for reinforcing polymer composites [28,29,30]. Because of their remarkable qualities, CNTs are used in various specialized engineering applications, which has caught the interest of many researchers [31,32]. Single-wall or multiple-wall CNT structures are both present [33]. Single-walled carbon nanotubes are made by rolling a single graphene sheet into a cylinder 1 nm in diameter and centimeters long [17]. It is stated in the literature that the improvement in the mechanical behavior of CNTRCs is limited when the CNTs are uniformly distributed [34,35]. There are several studies performed in this field. Ong et al. [36] investigated the dynamic responses of the double-beam system, in which CNTs are used to reinforce the structure with different dispersion patterns. Zhu et al. [37] investigated the effect of the CNT dispersion model on bending deformation using finite element methods. The review papers by Sharma and Joshi [38] and Zaghloul et al. [39] may be referred to for an extensive literature survey of FG-CNTRC structures.

Many studies on the mechanical characteristics of CNTs were conducted. The elastic modulus of composites based on CNTs has been experimentally measured, and it is shown to be higher than that of the pure matrix. The addition of CNTs has been reported to enhance the mechanical aspects of polymer-based composites significantly. Tarfaoui et al. [40] employed various volume fractions of CNTs in a fabric made of carbon fibers and epoxy. They calculated that 0.5% to 2% of CNT-reinforced textile composites are required for the crucial volume fraction barrier. They explained that a 4% decrease in mechanical behavior, the impact of CNT dispersion, and the existence of an upper limit begin at 2%. Qian et al. [41] were intrigued by the finding that adding a small percentage of CNTs (approximately 1 wt%) to a matrix material can boost the composite stiffness by 36% to 42% and tensile strength by 25%. Gouda et al. [42] investigated a hybrid polymer composite that was filled with CNTs (0.2 wt%) and graphene (0.2 wt%). To determine whether an instrumented indentation approach might be used to measure the mechanical characteristics of nanocomposites, Lee et al. [43] examined three different types of CNT-reinforced composites. Makvandi and Öchsner [44] discovered that the placement of the short fibers had a significant impact on the composite’s elastic modulus and stiffness. The findings showed that nanocomposite flexural strength increases when CNTs are chemically linked to glass fibers, while their tensile strength is marginally reduced.

Theoretical and numerical investigations were performed to ascertain the impact of adding CNTs on the material characteristics and resistance of composite materials. Madenci [45] created a mixed FEM model to perform free vibration assessments of functionally graded CNTRC nanobeams, based on the trigonometric shear deformation theory. Single-walled CNTs were assumed to be distributed uniformly and functionally graded along the thickness directions in each layer of the perfectly bonded CNTRC beam. The practical material characteristics of CNTRCs are determined using the mixture rule, where the CNT efficiency parameters are integrated to account for the scale dependence of the resulting nanostructures. Vinyas [46] investigated the coupled nonlinear deflections of multiphase magneto-elastic plates reinforced with CNT. A finite element method, including nonlinear strain displacement relation and Reddy’s higher-order shear deformation theory, was used to create the governing equations. Mehrabadi et al. [47] concluded that functionally graded CNTRC plates with symmetric distribution profiles are a potential replacement for plates with uniformly distributed CNTs. Based on first and high-order shear deformation beam theories, Lin and Xiang [48] suggested the vibration characteristics of CNTRC beams. Di Sciuva and Sorrenti [49] investigated the bending, free vibration and buckling analysis of functionally graded CNTRC plates using extended refined zigzag theory.

This paper investigates the influence of CNTs, including polymer mix-based composites, on bending analysis with analytical and experimental approaches. Small CNT concentrations can significantly change the mechanical behavior of composites. In the case of smaller CNT weight fractions, significant improvement has been attained. Within the scope of the study, neat epoxy and 0.3% by weight CNTRC carbon fiber composite samples were prepared. The experimental tensile and bending tests were utilized to obtain the mechanical properties of the CNTRC beam. The mechanical properties such as density and Young’s moduli are obtained through the experimental tests. Micromechanical modelling was also used to obtain the mechanical properties. An analytical solution was made to confirm the experimental results. The analytical solution of bending analysis was applied using the Timoshenko beam theory. The governing equations of motion are derived using Hamilton’s principle for mentioned theories. In addition to the experimental tests, calculations are made with the mixture-rule model.

## 2. Experimental Program

In this experimental program, tensile and bending tests were utilized. The samples with 0% (NEAT) and 0.3% CNT were tested in these experiments. For tensile tests, two repetitions were performed, while three repetitions were performed for the bending tests.

### 2.1. Materials

**Figure 1:**
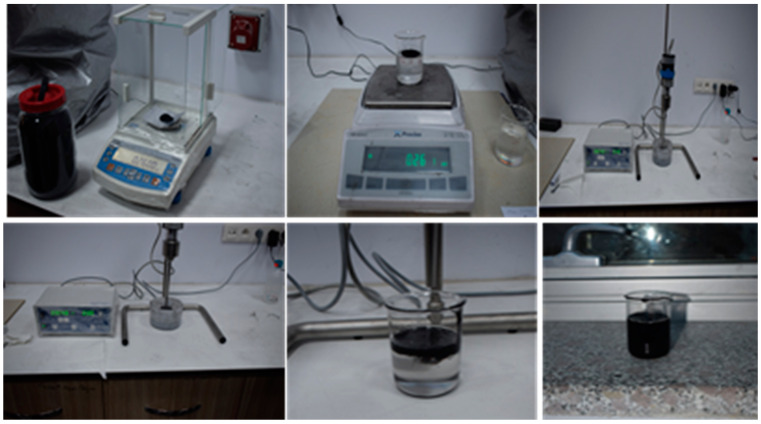
CNTs in epoxy resin and using the mixture of resin/CNT as a matrix of the composite.

**Figure 2:**
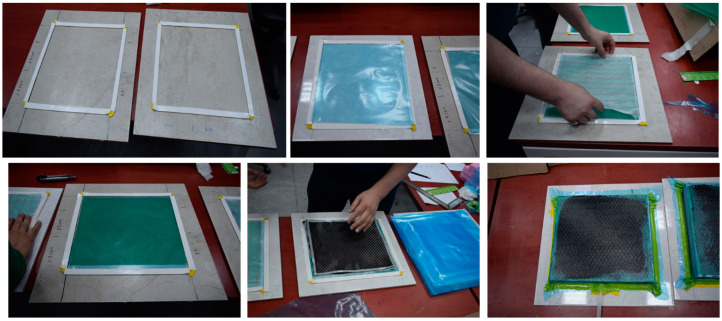
Manufacture of CNTRC composite material.

This study produced epoxy matrix carbon fiber reinforced composite plates by symmetrically laying three layers of fabric on the mold using the vacuum infusing method and sending resin into it. While producing the composite plates, they were left to cure at room temperature for 24 h. The same method is used for nanoparticles consisting of three layers of carbon fiber fabric. At this stage, MWCNTs were incorporated into the epoxy matrix at a rate of 0.3% by weight. In the first step, the powder of CNTs was mixed with epoxy resin using a mechanical stirrer for 15 min. In the second step, an ultrasonic homogenization device was used after using a mechanical stirrer. The dispersion process was carried out with an ultrasonic homogenizer device at a power rate of 15% and for 15 min (Figure 1). This step was applied to achieve the best possible dispersion of the powder of carbon nanotubes in the epoxy resin. Before starting the second step again, the mixture was kept in a cooled ultrasonic bath for 15 min to lower the temperature of the nano-reinforced resin resulting from the kinetic energy of the particles produced by the ultrasonic mixer tip. In the final step, the nano-reinforced resin was injected into the carbon fiber fabric by a vacuum infusion process (Figure 2).

Tensile and three-point bending tests were performed on carbon fiber-reinforced thermoset matrix composite specimens to examine the studies and evaluate the results. The mechanical properties of these materials are shown in Table 1.

### 2.2. Tensile Tests

The samples with nominal dimensions of 250 × 25 × 3 mm (length-width-thickness) were tested under tensile forces to determine their tensile strength. The gap between the gages measured 200 mm. The ASTM D3039 procedure was followed. The forces were applied at a rate of one millimeter per minute.

### 2.3. Bending Tests

**Figure 3:**
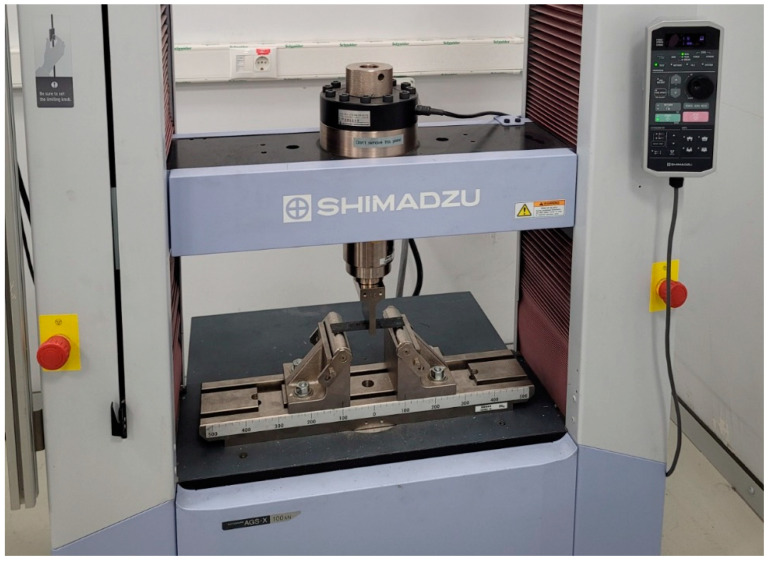
Three-point bending test setup.

Three-point bending loads were applied during testing to ascertain the flexural capability of the samples. The procedure of ASTM D7264 was applied. The specimens’ nominal measurements are 150 × 25 × 3 mm. The effective span was 100 mm. The same testing device was used to evaluate the samples. The loading speed for the bending tests was 1 mm/min, the same as the tensile test. The test configuration for the bending test is depicted in Figure 3.

## 3. Analytical Formulation

For the calculation of the mechanical behavior of CNTRCs under buckling stress, it is necessary to know Young’s modulus, Poisson’s ratio, and shear modulus. In this section, the theoretical mixture-rule model used to calculate composite mechanical properties is defined as

(1) $$E_{11}=\eta_{1}V_{CNT}E_{11}^{CNT}+V_{m}E_{m}$$

(2) $$\frac{\eta_{2}}{E_{22}}=\frac{V_{CNT}}{E_{22}^{CNT}}+\frac{V_{m}}{E_{22}^{m}}$$

(3) $$\frac{\eta_{3}}{G_{12}}=\frac{V_{CNT}}{G_{12}^{CNT}}+\frac{V_{m}}{G_{m}}$$

(4) $$\nu_{12}=V_{CNT}\nu_{12}^{CNT}+V_{m}\nu_{m}$$

(5) $$\rho =V_{CNT}\rho_{CNT}+V_{m}\rho_{m}$$

where “$E_{11}^{CNT}$, $E_{22}^{CNT}$ and $G_{12}^{CNT}$” are the Young’s modulus and shear modulus of the single-walled CNT, respectively. “$E_{m}$ and $G_{m}$” represent the isotropic matrix’s corresponding properties. To account for scale-dependent material properties, “$\eta_{i}(i=1,2,3)$” are CNT/matrix efficiency parameters that can be determined by matching the effective properties of CNTRC observed in molecular dynamics simulations with the extended rule of mixture predictions. “$\rho_{CNT}$” and “$\rho_{m}$” are the CNT and matrix mass densities, respectively. “$V_{CNT}$” and “$V_{m}$” are the volume fractions of the CNT and matrix, which are related by

(6) $$V_{CNT}+V_{m}=1$$

The mixture rule can be used to compute the Young’s modulus of a unidirectional lamina in the longitudinal direction and the Poisson’s ratio. The following mixture rule is created for the mechanical characteristics of the fiber and matrix:

(7) $$E_{11}=V_{fiber}E_{11}^{fiber}+V_{m}E_{m}$$

(8) $$\nu_{12}=V_{fiber}\nu_{12}^{fiber}+V_{m}\nu_{m}$$

Consider a multilayer CNTRC beam made up of “*N*” layers with identical thicknesses “*t*” and having a length “*L*” and total thickness “*h*”. According to the Timoshenko beam theory, the displacement field is made up of the axial displacement “*u*” and the transverse displacement “*w*”, and it takes the following forms:

(9) $$\begin{array}{l} u(x,z,t)=u_{0}(x,t)+z\phi_{x}(x,t)\\ v(x,z,t)=0\\ w(x,z,t)=w_{0}(x,t) \end{array}$$

where “$u_{0}$” and “$w_{0}$” refer to the axial and transverse displacements of the beam, respectively, in its mid-plane (*z* = 0). The transverse normal’s mid-plane rotation about the y-axis is known as the “$\phi_{x}$”. It is possible to assess the strain-displacement relations of the CNTRC beam as:

(10) $$\begin{array}{l} \varepsilon_{xx}=\frac{\partial u_{0}}{\partial x}+z\frac{\partial \phi_{x}}{\partial x}\\ \gamma_{xz}=\frac{\partial w_{0}}{\partial x}+\phi_{x} \end{array}$$

One way to express Hamilton’s premise is as

(11) $$\int_{t_{1}}^{t_{2}}\left(\delta U+\delta V-\delta K\right)dt=0$$

“*K*” stands for the kinetic energy represented by

(12) $$\begin{array}{l} \delta K=\int_{0}^{L}\int_{A}\rho (z)\left[u\delta u+w\delta w\right]dAdx\\ \;=\int_{0}^{L}\left[I_{0}(u_{0}\delta u_{0}+w_{0}\delta w_{0})+I_{1}(\phi_{x}\delta u_{0}+u_{0}\delta \phi_{x})+I_{2}\phi_{x}\delta \phi_{x}\right]\;dx \end{array}$$

in which the mass moment of inertia specified by “*I_i_*(*i* = 0, 1, 2)”

(13) $$I_{i}=\int_{A}\rho (z)z^{i}dA\;\;\;(i=0,1,2)$$

where “δU” represents the entire strain energy’s virtual variation

(14) $$\begin{array}{l} \delta U=\int_{0}^{L}\int_{A}\left(Q_{11}\varepsilon_{xx}\delta \varepsilon_{xx}+Q_{55}\gamma_{xz}\delta \gamma_{xz}\right)dAdx\\ \;=\int_{0}^{L}\left(N_{x}\frac{du_{0}}{dx}-M_{x}\frac{d\delta \phi_{x}}{dx}+Q_{x}(\frac{d\delta w_{0}}{dx}-\delta \phi_{x})\right)dx \end{array}$$

and “δV” represents the virtual work completed by the axially compressive force “$N_{x0}$” and transverse load “*q*” as described by

(15) $$\delta V=-\int_{0}^{L}\left(q\delta w_{0}+N_{x0}\frac{\partial w_{0}}{\partial x}\frac{\partial \delta w_{0}}{\partial x}\right)dx$$

where the stress-related effects are as follows

(16) $$\begin{array}{l} N_{x}=\int_{A}\sigma_{xx}dA\\ M_{x}=\int_{A}\sigma_{xx}zdA\\ Q_{z}=\int_{A}\sigma_{xz}dA \end{array}$$

The simplified stress-strain constitutive equations are:

(17) $$\begin{array}{l} \sigma_{xx}=Q_{11}\varepsilon_{xx}\\ \sigma_{xz}=Q_{55}\gamma_{xz} \end{array}$$

in where

(18) $$\begin{array}{l} Q_{11}=\frac{E_{1}}{1-\vartheta_{12}\vartheta_{21}}\\ Q_{55}=G_{13} \end{array}$$

Substituting Equations (10) and (17) into Equation (16), all stress resultants can be written in the form of material stiffness components and displacements as follows:

(19) $$\begin{array}{l} \frac{\partial \phi_{x}}{\partial x}=D_{11}^{*}M_{x}\\ \frac{\partial w_{0}}{\partial x}+\phi_{x}=\frac{1}{k_{s}}A_{55}^{*}Q_{x} \end{array}$$

where “ks=56” is the shear correction factor and “$D_{11}^{*}$ and $A_{55}^{*}$” are its inverse matrix’s components.

(20) D11=∫−h2h2Q11z2dz=∑k=1N∫zkzk+1Q11(k)z2dzA55=∫−h2h2Q55 dz=∑k=1N∫zkzk+1Q55(k)dz

The following equations of motion for symmetrically laminated beams are obtained by using the integration by parts approach and gathering the coefficients of “$\delta w_{0}$” and “$\delta \varphi_{x}$” when the in-plane displacements “$u_{0}$” are zero.

(21) $$\begin{array}{l} \frac{\partial Q_{x}}{\partial x}+{\hat{N}}_{x0}\frac{\partial^{2}w_{0}}{\partial x^{2}}+q=I_{0}\frac{\partial^{2}w_{0}}{\partial t^{2}}\\ \frac{\partial M_{x}}{\partial x}-Q_{x}=I_{{}_{2}}\frac{\partial^{2}\phi_{x}}{\partial t^{2}} \end{array}$$

The equations of motion Equation (19) can be recast using Equation (21), in terms of the displacements, as follows:

(22) $$\begin{array}{l} k_{s}G_{xz}A(\frac{\partial^{2}w_{0}}{\partial x^{2}}+\frac{\partial \phi_{x}}{\partial x})+b{\hat{N}}_{x0}\frac{\partial^{2}w_{0}}{\partial x^{2}}+\hat{q}={\hat{I}}_{0}\frac{\partial^{2}w_{0}}{\partial t^{2}}\\ E_{xx}I_{yy}\frac{\partial^{2}\phi_{x}}{\partial x^{2}}-k_{s}G_{xz}A(\frac{\partial^{2}w_{0}}{\partial x}+\phi_{x})={\hat{I}}_{2}\frac{\partial^{2}\phi_{x}}{\partial t^{2}} \end{array}$$

The inertia terms are disregarded, and the axial force is set to zero for the static bending problem. Equation (22) then becomes:

(23) $$k_{s}G_{xz}bh\left(\frac{d^{2}w_{0}}{dx^{2}}+\frac{d\phi_{x}}{dx}\right)+\hat{q}=0$$

$$E_{xx}I_{yy}\frac{d^{2}\phi_{x}}{dx^{2}}-k_{s}G_{xz}bh\left(\frac{dw_{0}}{dx}+\phi_{x}\right)=0$$

where “$\hat{q}=bq$”.

Integrating Equation (23) in relation to x

(24) $$k_{s}G_{xz}bh\left(\frac{dw_{0}}{dx}+\phi_{x}\right)=-\int_{0}^{x}\hat{q}(\xi )d\xi +C_{1}$$

The following can be obtained by integrating with respect to *x* and substituting the result into Equation (23): [50]

(25) $$E_{xx}I_{yy}\frac{d\phi_{x}}{dx}=-\int_{0}^{x}\int_{0}^{\eta }\hat{q}(\xi )d\xi d\eta +C_{1}x+C_{2}$$

(26) $$E_{xx}I_{yy}\phi_{x}(x)=-\int_{0}^{x}\int_{0}^{\zeta }\int_{0}^{\eta }\hat{q}(\xi )d\xi d\eta d\zeta +C_{1}\frac{x^{2}}{2}+C_{2}x+C_{3}$$

Substituting for $\phi_{x}(x)$ from Equation (25) into Equation (24) [50]

(27) $$\frac{dw_{0}}{dx}=-\frac{1}{E_{xx}I_{yy}}\left[-\int_{0}^{x}\int_{0}^{\zeta }\int_{0}^{\eta }\hat{q}(\xi )d\xi d\eta d\zeta +C_{1}\frac{x^{2}}{2}+C_{2}x+C_{3}\right]+\frac{1}{k_{s}G_{xz}bh}\left[-\int_{0}^{x}\hat{q}(\xi )d\xi +C_{1}\right]$$

(28) $$\begin{array}{ll} w_{0}(x)= & -\frac{1}{E_{xx}I_{yy}}\left[-\int_{0}^{x}\int_{0}^{\zeta }\int_{0}^{\eta }\int_{0}^{\mu }\hat{q}(\zeta )d\zeta d\mu d\eta d\xi +C_{1}\frac{x^{3}}{6}+C_{2}\frac{x^{2}}{2}+C_{3}x+C_{4}\right]\\ & +\frac{1}{k_{s}G_{xz}bh}\left[-\int_{0}^{x}\int_{0}^{\xi }\hat{q}(\zeta )d\zeta d\xi +C_{1}x\right] \end{array}$$

## 4. Results

### 4.1. Tension Test Results

**Figure 4:**
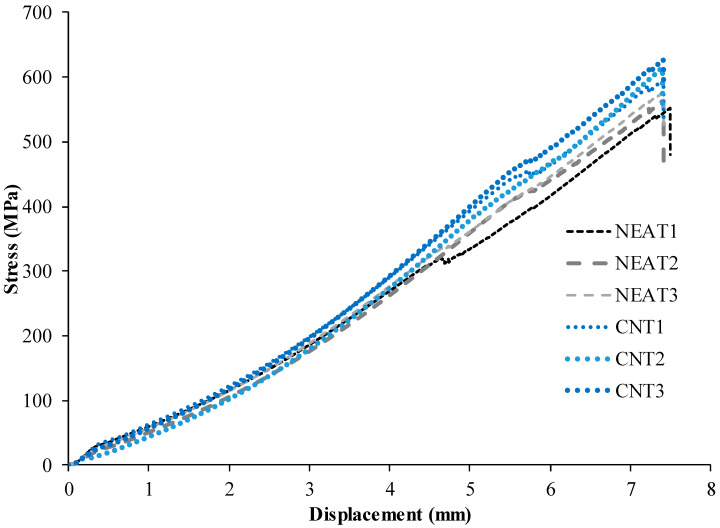
Tensile test results of the samples.

Load-displacement curves of the axial tension test are depicted in Figure 4. The results demonstrated that the average tensile stress of CNT1, CNT2, and CNT3 at failure was 612 MPa, while for NEAT1, NEAT2, and NEAT3, it was 564 MPa. The maximum tensile stress values of CNT1, CNT2, and CNT3 were 5.7%, 8.8% and 11.1% higher than the averages of NEAT1, NEAT2, and NEAT3, respectively (Table 2). Therefore, it was determined that the samples with CNT carried 8.4% more axial tensile force on average than those with NEAT. All specimens exhibited linear elastic behavior and collapsed as soon as they reached a displacement of about 7.40 mm. However, when Figure 4 is examined, the samples with CNT have reached the same failure displacement with higher load-carrying capacities. The stress values are also depicted in Figure 4. The initial stiffness values, that is, the angle of the curve with the horizontal, were obtained more in the samples with CNT after the 4 mm displacement value.

### 4.2. Bending Test Results

**Figure 5:**
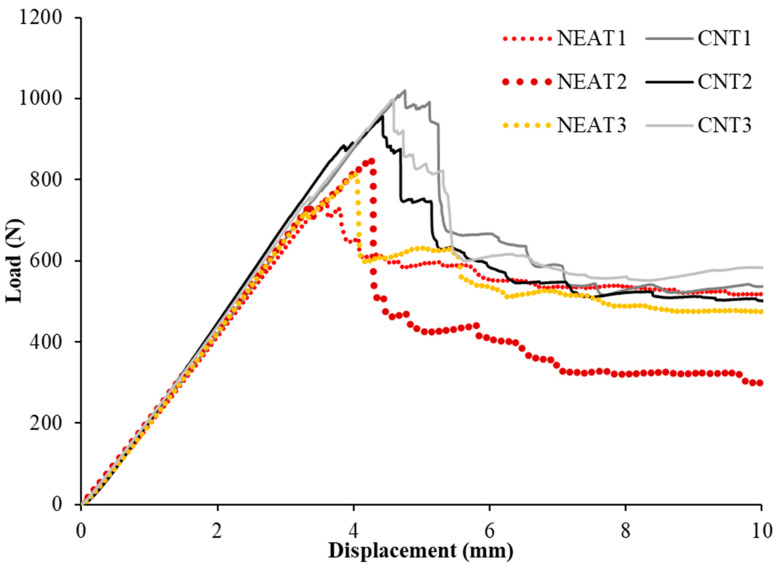
Bending test results.

The load-displacement relationships of the samples under bending are shown in Figure 5. Flexural load carrying capacities were obtained as 1020 N, 956 N, and 998 N for CNT1, CNT2, and CNT3, respectively, while 752 N, 849 N, and 814 N were obtained for NEAT1, NEAT2, and NEAT3 samples, respectively. In this case, the CNT1 sample has 35.6%, 20.1%, and 25.3% more bending capacity than those of NEAT1, NEAT2, and NEAT3, respectively. A similar situation was obtained for CNT2 at 27.1%, 12.6%, and 17.4%, while for CNT3, it was 32.7%, 17.5%, and 22.6% (Table 3). In other words, the samples with CNT had an average of 23.4% more bending capacity than the samples with NEAT. The displacement values corresponding to the maximum load-carrying capacity of all samples varied between 3.57 mm and 4.75 mm. This situation can be explained by the variation of the stiffness values corresponding to the maximum load value. When the stiffness of the samples at the maximum load value were compared, a relationship such as CNT3 > CNT2 > CNT1 > NEAT1 > NEAT2 > NEAT3 was obtained. This shows that the stiffness of the CNT specimens improved along with their load-carrying capacity.

**Figure 6:**
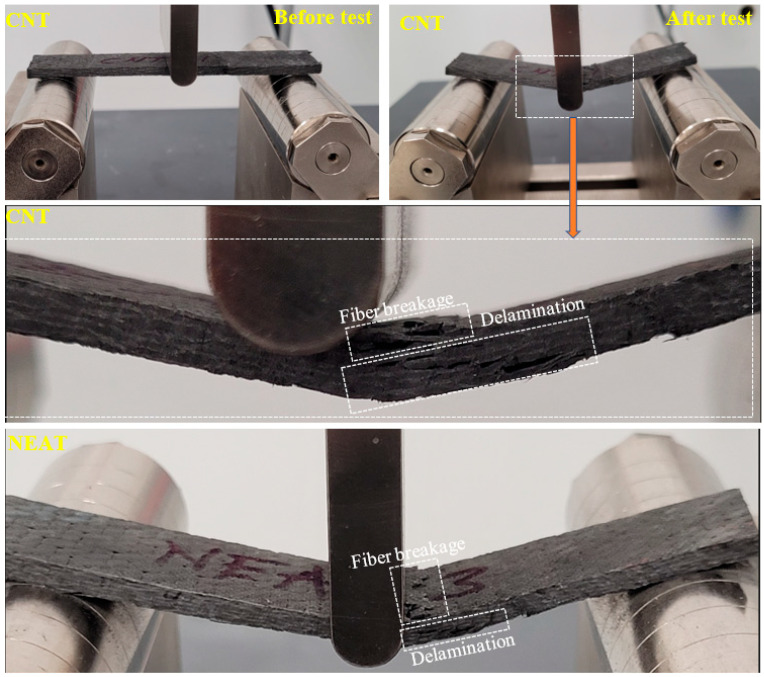
Damage analysis of samples.

The damage modes on the samples are shown in Figure 6. The ultimate damage observed for all samples is delamination damage along the sample’s height (thickness = 3 mm) and fiber breakage in the compression region.

Compared to the experimental results, the material properties of the samples made using micro-mechanic models and displacements were measured analytically, and the results are shown in Table 4 and Table 5.

According to the results, CNT-reinforced samples exhibited an average tensile strength of 612 MPa and a tensile modulus of 14.42 GPa, while pure epoxy samples exhibited a tensile strength average of 563 MPa and tensile modulus of 13.18 GPa. Adding 0.3% by weight of MWCNT increases tensile modulus by approximately 9%.

## 5. Conclusions

This research examines the effects of adding CNTs to conventional polymer mix-based composites. The experimental measurement of elastic moduli of composites-based CNTs shows that there is an increase in the composite modulus over the pristine matrix modulus [40]. In the experimental and analytical study, the following results can be expressed.

- Small CNT concentrations can significantly change the mechanical behavior of composites. Lower CNT weight fractions have resulted in a significant improvement.
- The flexural and tensile strength was improved in the presence of CNTs, proving that they can be utilized as a support material. In other words, the flexural strength and flexural modulus increase when 0.3% by weight of MWCNT is added. This positive effect can be explained by the fact that carbon nanotubes prevent crack opening and propagation by bridging cracks during fracture. By producing nanocomposites and examining their mechanical properties, Zheng et al. [51] obtained similar results to this study. Firstly, they examined the effects of nanoparticles on the epoxy matrix and found different reinforcements by weight. The best increase in the composites they prepared was at 0.3%; the tensile strength, tensile modulus and impact strength with the addition of nanoparticles obtained were 115%, 13% and 60%, respectively. One-way glass fiber reinforced polymer produced a 69.4% increase in flexural strength, tensile modulus and strength; an increase of 21% and 23% was determined, respectively. In this study, adding 0.3% by weight of MWCNT increased the tensile strength and tensile modulus by approximately 11% and 12%, respectively.
- For upcoming applications requiring high mechanical performance, CFRP composites loaded with MWCNTs are anticipated to be quite promising, given the increases in the samples’ flexural modulus and strength.

## Figures and Tables

**Table 1:** Material properties.

Parameters	Carbon Fiber	Epoxy Matrix		CNT
*E*_11_ (GPa)	230	2.72	*E* (GPa)	500
*E*_22_ (GPa)	15	2.72	*v*	0.26
*E*_33_ (GPa)	15	2.72	*t* (nm)	0.34
*v*_12_	0.28	0.3	*l* (m)	25 × 10^−6^
*v*_13_	0.28	0.3	*d* (nm)	1.4
*v*_23_	0.28	0.3	*ρ* (kg/m^3^)	1350
*G*_12_ (GPa)	15	1.18		
*G*_13_ (GPa)	15	1.18		
*G*_23_ (GPa)	15	1.18		

**Table 2:** The maximum axial tension load and displacements of samples.

Sample	Maximum Stress (MPa)	Rate of Increase (%)	Maximum Displacement (mm)
NEAT1	552	⸻	7.49
NEAT2	563	⸻	7.41
NEAT3	576	⸻	7.41
CNT1	596	5.7%	7.41
CNT2	614	8.8%	7.40
CNT3	626	11.1%	7.40

**Table 3:** The maximum bending load and vertical displacements of samples.

Sample	Maximum Load (N)	Rate of Increase (%)	Displacement at Maximum Load (mm)	Rigidity at Maximum Load (N/mm)
NEAT1	752	⸻	3.57	210.66
NEAT2	849	⸻	4.27	198.86
NEAT3	814	⸻	4.06	200.62
CNT1	1020	35.6, 20.1 and 25.3	4.75	214.74
CNT2	956	27.1, 12.6 and 17.4	4.41	216.80
CNT3	998	32.7, 17.5 and 22.6	4.59	217.54

**Table 4:** Results of the Young’s modulus model.

Sample	Young’s Moduli (GPa)		Error (%)
	Experimental	Analytical	
0.0%wt CNT	13.18 ± 0.21	14.48	9%
0.3%wt CNT	14.42 ± 0.14	16.18	12%

**Table 5:** Results of the displacements.

Sample	Displacements (mm)		Error(%)
	Experimental	Analytical	
0.0%wt CNT	3.96 ± 0.29	4.10	4%
0.3%wt CNT	4.58 ± 0.14	4.63	1%

## Data Availability

Not applicable.
